# Non-Invasive Brain Stimulation in Conversion (Functional) Weakness and Paralysis: A Systematic Review and Future Perspectives

**DOI:** 10.3389/fnins.2016.00140

**Published:** 2016-03-31

**Authors:** Carlos Schönfeldt-Lecuona, Jean-Pascal Lefaucheur, Peter Lepping, Joachim Liepert, Bernhard J. Connemann, Alexander Sartorius, Dennis A. Nowak, Maximilian Gahr

**Affiliations:** ^1^Department of Psychiatry and Psychotherapy III, University of UlmUlm, Germany; ^2^Department of Physiology, Henri Mondor Hospital, Assistance Publique – Hôpitaux de ParisCréteil, France; ^3^EA 4391, Nerve Excitability and Therapeutic Team, Faculty of Medicine, Paris Est Créteil UniversityCréteil, France; ^4^Department of Psychiatry, Betsi Cadwaladr University Health BoardWrexham, UK; ^5^Centre for Mental Health and Society, Bangor UniversityWrexham, UK; ^6^Department of Psychiatry, Mysore Medical College and Research InstituteMysore, India; ^7^Department of Neurorehabilitation, Kliniken SchmiederAllensbach, Germany; ^8^Department of Psychiatry and Psychotherapy, Medical Faculty Mannheim, Central Institute of Mental Health, University of HeidelbergMannheim, Germany; ^9^Department of Neurology, Helios-Klinik KipfenbergKipfenberg, Germany; ^10^Department of Neurology, University Hospital MarburgMarburg, Germany

**Keywords:** hysterical paralysis, hysterical neuroses, medically unexplained motor symptoms, functional neurological disorder, functional lesion, psychogenic movement disorders, magnetic stimulation, electroshock

## Abstract

Conversion (functional) limb weakness or paralysis (FW) can be a debilitating condition, and often causes significant distress or impairment in social, occupational, or other important areas of functioning. Most treatment concepts are multi-disciplinary, containing a behavioral approach combined with a motor learning program. Non-invasive brain stimulation (NIBS) methods, such as electroconvulsive therapy (ECT), and transcranial magnetic stimulation (TMS) have been used in the past few decades to treat FW. In order to identify all published studies that used NIBS methods such as ECT, TMS and transcranial direct current stimulation (tDCS) for treating FW patients a systematic review of the literature was conducted in PubMed and Web of Science. In a second step, narratives were used to retrospectively determine nominal CGI-I (Clinical Global Impression scale–Improvement) scores to describe approximate changes of FW symptoms. We identified two articles (case reports) with ECT used for treatment of FW, five with TMS with a total of 86 patients, and none with tDCS. In 75 out of 86 patients treated with repetitive (r)TMS a nominal CGI-I score could be estimated, showing a satisfactory short-term improvement. Fifty-four out of seventy-five identified patients (72%) had a CGI-I score of 1 (very much improved), 13 (17%) a score of 2 (much improved), 5 (7%) a score of 3 (minimally improved), and 3 (5%) remained unchanged (CGI-I = 4). In no case did patients worsen after rTMS treatment, and no severe adverse effects were reported. At follow-up, symptom improvement was not quantifiable in terms of CGI-I for the majority of the cases. Patients treated with ECT showed a satisfactory short-term response (CGI-I = 2), but deterioration of FW symptoms at follow-up. Despite the predominantly positive results presented in the identified studies and satisfactory levels of efficacy measured with retrospectively calculated nominal CGI-I scores, any assumption of a beneficial effect of NIBS in FW has to be seen with caution, as only few articles could be retrieved and their quality was mostly poor. This article elucidates how NIBS might help in FW and gives recommendations for future study designs using NIBS in this condition.

## Introduction

Conversion Disorder is a frequent condition. It is classed under “dissociative and conversion disorders” in the international WHO-classification (WHO ICD-10, 1991) and “Functional Neurological Symptom Disorder (FNS)” in DSM-5 (DSM-5, 2013). The precise prevalence of the disorder is unknown. The reported incidence is between 4 and 12 cases per 100,000 habitants/year (DSM-5, 2013). In the largest prospective cohort study, conversion disorder accounted for 5.6% of 3781 Scottish patients referred from primary care to a National Health Service neurology clinic (Stone et al., 2009).

Conversion (functional) weakness or paralysis (FW) [DSM-5 300.11/ICD-10 F44.4], a subgroup of FNS that affect limbs, can be very incapacitating and causes significant distress or impairment in social, occupational, or other important areas of functioning (Table 1 for DSM-5 criteria). In FW, symptoms either cannot be explained by a neurological condition (or other general medical condition), or clinical findings are inconsistent with recognized neurological or medical disease (DSM-5, 2013). Therefore, in the literature, such disorders have been referred to as “psychogenic,” “hysterical,” “non-organic”. or rather unfortunately, “pseudo-neurological” (Nowak and Fink, 2009). The underlying etiological mechanisms involved remain unclear. Psychological factors were required in DSM-IV (former criterion B: “Psychological factors are judged to be associated with the symptom or deficit because the initiation or exacerbation of the symptom or deficit is preceded by conflicts or other stressors”; Carson et al., 2012). This criterion has been removed in DSM-5. Although conflicts and stressors may influence patients' vulnerability there is increasing evidence for a neurobiological component in the etiology of FW (Liepert et al., 2008, 2009, 2011). Over the last decade, neuroimaging findings examining differential brain activity in FW have started to support a neuro-biological hypothesis (Marshall et al., 1997; Spence et al., 2000; Vuilleumier et al., 2001; Vuilleumier, 2005; Burgmer et al., 2006; Stone et al., 2007; de Lange et al., 2008; Cojan et al., 2009; van Beilen et al., 2011; Ludwig et al., 2015), for review (Nowak and Fink, 2009). Even if the disorder is sometimes not easy to differentiate from simulation or malingering in a phenomenological way, FW is different from a neurobiological point of view and shares similarities with hypnotically induced paralysis (Bell et al., 2011; Ludwig et al., 2015).

**Table 1:** **DSM-5 diagnostic criteria for Conversion Disorder (Functional Neurological Symptom Disorder/FNS)**.

A. One or more symptoms or altered voluntary motor or sensory function.
B. Clinical findings provide evidence of incompatibility between the symptom and recognized neurological or medical condition.
C. The symptom or deficit is not better explained by another medical or mental disorder.
D. The symptom or deficit causes clinically significant distress or impairment in social, occupational, or other important areas of functioning or warrants medical evaluation.
Coding note: The ICD-9-CM code for conversion disorder is 300.11, which is assigned regardless of the symptom type. The ICD-10-CM codes depends on the symptom type (see below).
*Specify symptom type:*
• (F44.4) With weakness or paralysis
• (F44.4) With abnormal movement (e.g., tremor, dystonic movement, myoclonus, gait disorder)
• (F44.4) With swallowing symptoms
• (F44.4) With speech symptoms (e.g., dysphonia, slurred speech)
• (F44.5) With attacks or seizures
• (F44.6) With anesthesia or sensory loss
• (F44.6) With special sensory symptoms
• (F44.7) With mixed symptoms
*Specify if:*
Acute episode: Symptoms present for < 6 months.
Persistent: Symptoms occurring for 6 months or more.
*Specify if:*
With psychological stressor *(specify stressor)*.
Without psychological stressor.

FW affecting limbs may be transient but can persist. The socio-economic disease-burden is significant because of direct treatment costs and the consequences of an often-permanent loss of limb function leading to incapacity-related benefits (Carson et al., 2011). In the past years, various treatment strategies have been tested in FW related symptoms, including different forms of physiotherapy (for review Nielsen et al., 2013), pharmacotherapy (Rampello et al., 1996; Voon and Lang, 2005), behavioral therapy (Shapiro and Teasell, 2004), and hypnotherapy (Moene et al., 2002). The reported symptom recovery is very heterogeneous and varies depending on the treatment strategy and study. A large amount of new studies reported marked short-term improvements, mostly in the region of a 60–70% symptom reduction (Nielsen et al., 2013). However, long-term outcome, especially in patients with a long duration of illness at presentation is invariably poor (Feinstein et al., 2001). Factors related to patient beliefs and disease concepts often generate difficulties in the treatment of FW. UK neurologists describe patients with FNS as being “the most difficult to help” (Carson et al., 2004). Although there is no agreement on the most effective therapy for FW, most treatment concepts contain at least two components: a behavioral approach and a motor learning program using a multidisciplinary team (Nielsen et al., 2013).

Non-invasive brain stimulation (NIBS) methods, such as electroconvulsive therapy (ECT), transcranial magnetic stimulation (TMS), and transcranial direct current stimulation (tDCS), have been used in the past decades to treat various mental disorders and may show beneficial effects in FW symptoms:

1. ECT, which was experimentally developed in the late 1930s (Cerletti, 1940) was the first NIBS method to become established within the framework of psychiatry. Based on an electrically induced generalized seizure ECT is used for the treatment of various mental disorders including affective and schizophrenia spectrum disorders, and is considered the most effective treatment in major depression (Taylor, 2008).
2. TMS is a non-convulsive NIBS method, which was initially developed for diagnostic purposes in order to measure motor latencies in the 1980s (Barker et al., 1985), and rapidly expanded in its repetitive form (rTMS) to a treatment strategy in the early 1990s. In 2010, the American FDA approved it for the treatment of therapy-resistant major depression in adults, although the clinical relevance of its efficacy remains doubtful (Schönfeldt-Lecuona et al., 2010; Lepping et al., 2014). The American Psychiatric Association (APA), the Canadian Network for Mood and Anxiety Treatments (CANMAT), and the World Federation of Societies of Biological Psychiatry (WFSBP) have accepted it as a treatment option for depression. It has been tested experimentally in other neuropsychiatric conditions (Lefaucheur et al., 2014).
3. tDCS is based on a homogeneous electrical field at direct current (DC) intensities of around 1 mA applied trans-cranially to accessible cortical areas (Nitsche and Paulus, 2011). tDCS induces long-lasting cortical changes and thus can be used to manipulate brain excitability via membrane polarization. The induced after-effects depend on polarity, duration and intensity of the stimulation (Paulus, 2011). tDCS is still an experimental treatment method in psychiatry but has demonstrated potential therapeutic efficacy in different conditions (Koops et al., 2015; Meron et al., 2015; Saba et al., 2015).

The exact mechanism of action of any of these NIBS methods on cortical networks is not yet comprehensively understood. However, it is known, that ECT facilitates the release of brain derived neurotrophic factor (BDNF) (Polyakova et al., 2015). It causes enlargement of hippocampal (and other) regions, possibly through boosting neurogenesis (Nordanskog et al., 2014). rTMS and tDCS have been shown to induce long-lasting changes in cortical excitability in directly stimulated cortical areas (Siebner and Rothwell, 2003; Powell et al., 2014; Romero Lauro et al., 2014) and in deeper interconnected brain areas (Strafella et al., 2003, 2004; Pogarell et al., 2007).

Measuring motor evoked potentials (MEP) using TMS was postulated for the first time to be advantageous in the management of FW patients by Jellinek et al. (1992). Using a figure-8 coil placed over the vertex, they performed MEPs of the first dorsal interosseus muscle for diagnostic purposes in a 25-year-old man with an acute functional flaccid paraplegia. MEPs of the paralyzed limb were within the normal range. One week after diagnostic TMS he experienced a full recovery. The authors associated the MEP-related muscular activation of the limbs with his recovery and argued that the patient's observation of the brisk (involuntary) limb contraction due to the cortical activation facilitated the successful symptom management. Schönfeldt-Lecuona et al. performed the first therapeutic rTMS trial in FW in 2003 in a patient suffering a right upper limb paralysis leading to a full and sustained recovery (Schönfeldt-Lecuona et al., 2003).

Our systematic review of the literature was conducted to identify all published studies that used NIBS methods for treating FW patients, and to discuss the potential of NIBS in this disorder. To achieve this we reviewed all published studies and reports (articles, published congress abstracts) of the use of TMS [in every modality: single-pulse(sp)TMS, rTMS including theta-burst protocol], tDCS and ECT in the treatment of FW affecting limbs.

## Methods

### Search strategy and selection criteria for the systematic review

A literature search was performed using PubMed and Web of Science databases with the below-elucidated search strategy. The literature search includes reports published until the 15 of December 2015. We defined search terms for the here explored forms of FW and NIBS methods. The following search terms were used for FW: “conversion disorder,” “motor conversion disorder,” “conversion weakness,” “conversion paralysis,” “dissociative weakness,” “dissociative paralysis,” “dissociative motor symptoms,” “dissociative ^*^ plegia,” “psychogenic disorder,” “psychogenic weakness,” “psychogenic paralysis,” “hysterical weakness,” “hysterical paralysis,” “hysterical conversion,” “hysterical ^*^ plegia,” “non-organic disorder,” “non-organic weakness,” “non-organic paralysis,” “non-organic ^*^ plegia,” “functional disorder,” “functional weakness,” “functional paralysis,” “functional ^*^ plegia,” “functional neurological symptoms,” and “medically unexplained neurological symptoms,” [^*^plegia, meaning all forms: mono-, hemi-, para-, tetra-, quadriplegia]. The following search terms were used for the different types of NIBS methods explained above: “stimulation,” “stimulation therapy,” “transcranial magnetic stimulation,” “TMS,” “rTMS,” “theta-burst^*^,” “transcranial direct current stimulation,” “tDCS,” “electroconvulsive,^*^” “electroshock,” and “ECT.”

**Figure 1:**
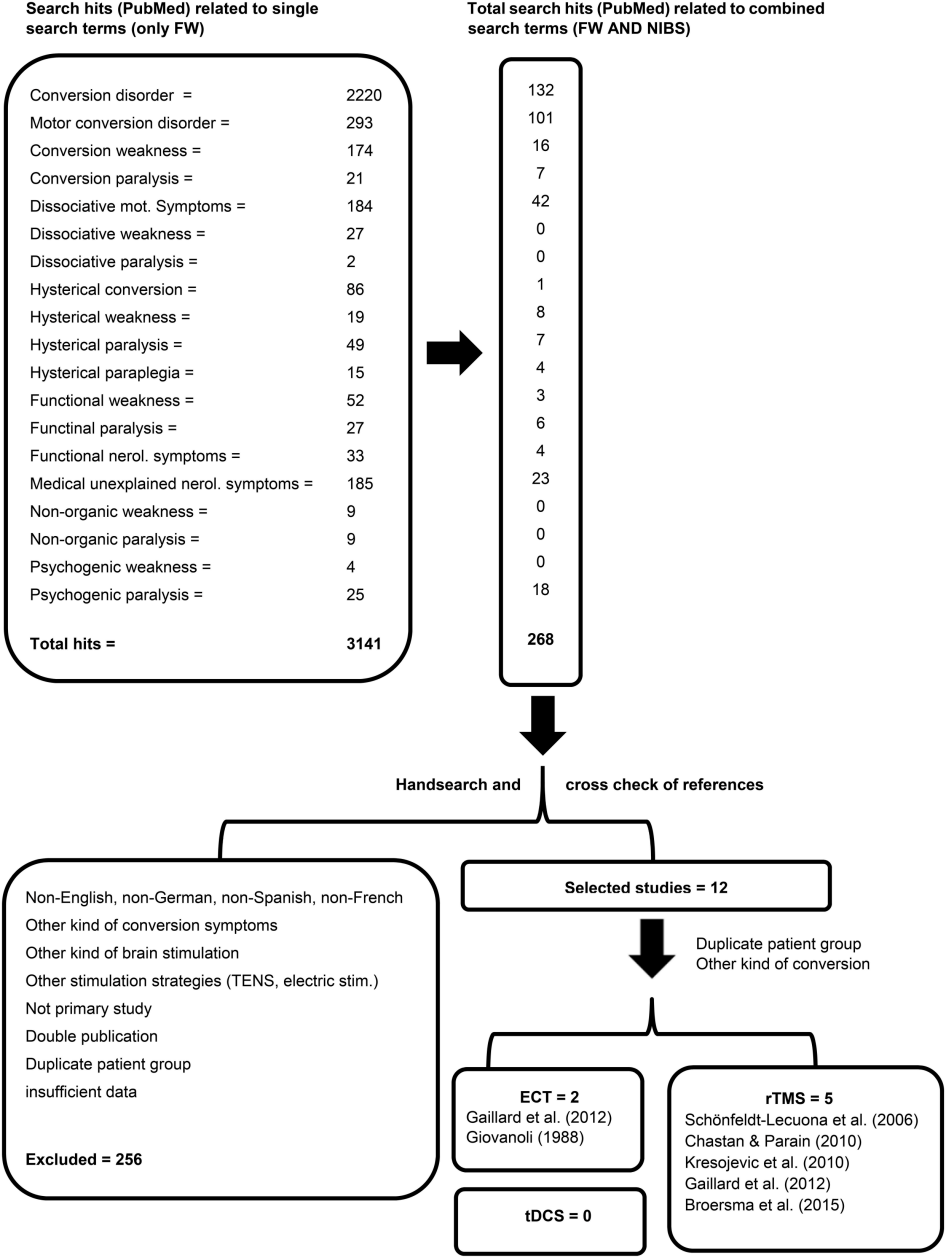
**The flowchart delineates the process and the results of the literature search**.

In a first step, the number of search hits related to each of the mentioned search terms for FW was retrieved (Figure 1). In a second step, each of the mentioned search terms related to FW was linked to all of the mentioned search terms (“AND”) related to the different types of NIBS methods (combined search), and the respective search hits were checked. Titles and abstracts related to the retrieved hits identified with the combined search were then checked manually by two examiners independently (CSL and MG, see below). In order to detect published conference and meeting abstracts edited in supplements not available in PubMed, a second independent search was carried out in Web of Science with the above mentioned search terms and then cross-checked. Because of space limitations, only the PubMed search results are shown in the Figure 1.

Inclusion criteria:

- Therapeutic trials only
- Patients exclusively suffering from FW as described above
- FW patients treated with TMS (in all variants: spTMS, rTMS including theta-burst TMS), tDCS or ECT
- Any kind of study design: randomized-controlled trials (RCT), non-RCT, open-label, naturalistic designs; all population sizes reported were allowed (full study, case series or case report)

Exclusion criteria:

- Non-English, non-German, non-Spanish, non-French language studies
- Conversion symptoms other than functional weakness or functional paralysis [non-FW as described here (DSM-5 300.11/ICD-10 F44.4), with or without sensory loss]
- Non therapeutic trial
- Non primary study, duplication, duplicated publication of data, duplicate patient group
- Insufficient data to evaluate treatment strategy and symptom outcome
- Disorders of consciousness presented as coma, vegetative state, minimally conscious state, stupor or catatonia.

Titles and abstracts of the articles retrieved from the combined search were checked for the presence of data of relevant topics (see above). Only articles addressing weakness or paralysis affecting limbs (often also accompanied by loss or reduction of sensory feeling) were considered. Two examiners (CSL and MG) then searched the retrieved titles and abstracts by hand and independently. After retrieval of abstracts fulfilling inclusion-criteria, full text versions of all identified articles were obtained. A cross check of the references from retrieved articles was performed to identify related publications not listed in the examined databases. Data were extracted independently by the two authors (CSL and MG). The two data bases were compared manually and then examined again by both reviewers. Discrepancies were corrected by reference to the original papers.

### Retrospective reconstruction of the clinical global impression - improvement (CGI-I) score

From all selected articles, the manuscript content was checked for clinical descriptions of symptom severity before and after treatment. The narratives were then used to determine an approximate change of FW symptoms, using the principles of the CGI-I scale for a nominal CGI-I score. Narratives were checked independently by two examiners (CSL and MG) and a nominal CGI-I score was established. In case of discrepancies, a consensus decision was reached between the two examiners. The CGI-I score is a 7-point scale which is commonly used to describe changes of a patient's clinical overall improvement related to a specific treatment. It was developed for use in NIMH (National Institute of Mental Health)-sponsored clinical trials to provide a brief, stand-alone assessment of the clinician's view of the patient's global functioning prior to and after initiating a study (Busner and Targum, 2007). CGI-I comprises the following categories: 1 = very much improved; 2 = much improved; 3 = minimally improved; 4 = no change; 5 = minimally worse; 6 = much worse; 7 = very much worse. If single data were available a nominal CGI-I was estimated for each patient reported (case reports and case series). In case of studies not reporting single data, a nominal CGI-I was calculated for the group of patients treated with a certain NIBS method.

## Results

On December 15, 2015, our literature search resulted in the following numbers of hits related to the different search terms: “conversion disorder” *n* = 2220, “motor conversion disorder” *n* = 293, “conversion weakness” *n* = 174, “conversion paralysis” *n* = 21, “dissociative motor symptoms” *n* = 184, “dissociative weakness” *n* = 27, “dissociative paralysis” *n* = 2, “hysterical conversion” *n* = 86, “hysterical weakness” *n* = 19, “hysterical paralysis” *n* = 49, “hysterical paraplegia” *n* = 15, “functional weakness” *n* = 52, “functional paralysis” *n* = 27, “functional neurological symptoms” *n* = 33, “medically unexplained neurological symptoms” *n* = 185, “non-organic weakness” *n* = 9, “non-organic paralysis” *n* = 9, “psychogenic weakness” *n* = 4, “psychogenic paralysis” *n* = 25. All in all, the literature search retrieved 3141 hits. The combined term search led to the results shown in the Figure 1. The search performed in Web of Science allowed the identification of one meeting abstract (Kresojevic et al., 2010) that was not identified using the PubMed database. We could not retrieve any other relevant publications using Web of Science, which were not identified using PubMed. Two articles reported the same patients and therefore had to be excluded (Schönfeldt-Lecuona et al., 2003; Broersma et al., 2013).

### Electroconvulsive therapy in FW

We identified *n* = 2 articles in which ECT was performed in FW (Table 2). In both peer-reviewed articles, a single case was reported (Giovanoli, 1988; Gaillard et al., 2012).

**Table 2:** **Electroconvulsive Therapy (ECT) and Transcranial Magnetic Stimulation (TMS) in functional weakness or paralysis (FW)**.

**Author (year) Nibs method-study type**	**Patients and clinical presentation**	**Stimulation protocol and technical data**	**Symptom development and efficacy (CGI-I)**	**Follow-up and other important issues**
Giovanoli, 1988 (ECT)-*Case report*	Fw pat (male, 61 years), right hand paralysis, 11 month prior to ect, after superficial laceration of middle finger	Ect on outpatient basis. Narcosis with thiopental sodium (50 mg), atropine (4 mg), and succinylcholine (10 mg). Bilateral ect (medcraft b-24), 3x/weekly for 2 weeks, then 2x/weekly for 6 weeks	Progressive improvement from the first ect in color and skin tone. 1 week after completion fine motor function of fingers restored (CCI rating), CGI-I = 2	After 6 month, and after 1 year patient was not using the hand any more but it was normal in appearance (CCI rating), CGI-I = 3
Gaillard et al., 2012 (ECT)-*Case report*	Fw pat (male 33 years), quadriplegia, 3 years prior to ECT	Initially 2–3 ects per week, modality (ns). Somewhat later 1 ect per week; than once a fortnight (in order to train motor skills and maintain mobility). Ect was performed at increasingly intensity until a maximum of 1152 mc in order to reach a seizure of at least 30–40 s	Until the 9th ect the progression in muscular activity allowed the patient to perform movements with increasingly complexity. He gained progressively more function and was able to eat without help, and to manage all activities of daily life in the perimeter of his room with only little help. Up to the 25th ect he was able to walk without help (CCI rating), CGI-I = 2	Relapse occurred after a while (ns), with great symptom fluctuation, dependent on the momentary circumstances, but muscular activity remained better than on admission (CCI rating), CGI-I = 3
Schönfeldt-Lecuona et al., 2006 (TMS)-*Case series, open-label*	3 FW pat. (1 male) + 1 malingerer. Age mv = 38 years; symptom duration: 5 weeks to 5 years	F8c, Dantec MacPro X 100, M1 stimulation, 4000 pulses/d, rTMS at 15Hz (2se train, ITI 8 sec), 5 times a week (working days); I = 110% MT for the first 2 weeks, then 90% MT for 4 to 12 weeks	All FW improved markedly (CCI rating) FW-Pat Nr. 1 CGI-I = 2 FW-Pat Nr. 2 CGI-I = 1 FW-Pat Nr. 3 CGI-I = 2	Improvement sustained at 6 and 12 months (CCI rating) FW-Pat Nr. 1 CGI-I = 1 FW-Pat Nr. 2 CGI-I = 1 FW-Pat Nr. 1 CGI-I = 2
Chastan and Parain, 2010 (TMS)-*Open-label, retrospective symptom assessment*	70 FW pat., age mv = 24.7 years (8–79); acute FW in 55 pat. (median duration 4 days); chronic FW in 15 pat. (median duration 240 days)	Cc, M1 stimulation, 30 pulses every 4 or 5 sec; 1 or 2 session in only 1 day, I = 100% maximal stimulator output	Immediately or within hours after rTMS effective in 89% of FW; ineffective in 11% (CCI rating) n = 53 pat. (75.7%) CGI-I = 1 n=9 pat. (12.8%) CGI-I = 2 n=5 pat. (7.2%) CGI-I = 3 n = 3 pat. (4.3%) CGI-I = 4	Effect sustained for the majority after 5 to 6 months. Recurrence of FW in 8 pat. In those pat., repeated rTMS was effective in 6 (CCI rating)
Kresojevic et al., 2010 (TMS)-*Case series*	1 FW pat. (male 24 years), “hemiparesis that compromised his walk.” Duration of symptoms (ns) 1 PMD pat. (not entered in the evaluation)	Cc, vertex stimulation, single rTMS session with 12 single pulses at initially 30% maximal stimulator output intensity and increasing I in 10% steps up to 80% of maximal stimulator output	“Immediate response, the pat. was able to walk again independently” (CCI rating) CGI-I = 2	Recurrence of mild symptoms after 6 months (partial deterioration), but mild walk difficulties did not influence his daily activities (CCI rating) CGI-I = 3
Gaillard et al., 2012 (TMS)-*Case report*	1 FW pat. male (33 years), quadriplegia, 6 months prior to rTMS	Coil type and I ns, rTMS at 1 Hz Fr. M1 stimulation, right and left over the arm-hand area, and right and left over legs” cortical motor area, 1000 pulses over each region (total = 4000 pulses per day), 5 times a week (working days, over a period of 8 weeks), after that, twice a week	Progressive amelioration: he was able to walk again, (rater impression, CGI-I = 1.5). Further deterioration led to a new rTMS treatment causing again symptom amelioration (CGI-I = 2.5), he was mobile only with a wheelchair. A third deterioration led to a new rTMS (CCI rating), CGII = 2	At follow-up recurrence of FW occurred (ns); he developed a phlebitis, pulmonary embolus and pressure soars, was referred for ECT (CCI rating), CGI-I = 4
Broersma et al., 2015 (TMS)-*placebo-controlled cross-over, single blinded*	11 FW pat. (4 male, 34-65 years), at least a flaccid hand paralysis; symptom duration: 4 weeks to 25 years	F8c, Magstim rapid2, contra-lateral M1 stimulation, 9000 pulses/d, rTMS at 15Hz (2setrain, ITI 4 sec), 5 times a week (working days) for 2 weeks; I = 80% MT (11 pat. received active, 8 pat. received placebo rTMS. Placebo rTMS with an electromagnetic device (REMP) placed in front of the magnetic coil at otherwise identical parameters	Primary outcome measure: muscle strength as measured by dynamometry; secondary outcome measure: subjective change in muscle strength; active rTMS induced a significantly larger median increase in objectively measured muscle strength (24%) compared to sham rTMS (6%); subjective ratings showed no statistical difference between treatments; no CCI rating	No follow-up data available.

pat., patient; F8c, figure-8-coil; Cc, Circular coil; mv, mean value; M1, motor cortex; MT, motor threshold intensity; Fr., Frequency; I, Intensity; d, day; s, seconds; (ns), no specified; PMD, psychogenic movement disorder; CCI, clinician's clinical impression; CGI-I, Clinical global impression scale-improvement item: 1 = very much improved; 2 = much improved; 3 = minimally improved; 4 = no change; 5 = minimally worse; 6 = much worse; 7 = very much worse.

#### Case description

Giovanoli (1988) presented a 61-year old man with a complete right hand paralysis after superficial laceration of the middle finger, 11 months duration prior to ECT. Bilateral ECT (Medcraft B-24) was performed on an outpatient basis 3-times per week for 2 weeks, then twice weekly for 6 weeks (ECT parameters not available). Within the first 10 ECT sessions, a progressive change in color and a decrease in swelling were observed; after the 10th ECT session, approximation of thumb and index finger was possible; after the 19th ECT edema had disappeared and the hand exhibited a full range of motion. One week after completion of ECT fine motor function of the patient's fingers was restored (he was able to button his shirt, tie his shoes and write). At 6 months follow-up and after 1 year, the patient did not use his hand during the examination but it was completely normal in appearance. No specific ECT parameters were stated. CGI-I after ECT was rated: 2 (much improved). The long-term CGI-I was rated: 3 (minimally improved).

Gaillard et al. (2012) presented a 33-year old man with fluctuating quadriplegia, developed 3 years prior to ECT. In total, 35 ECT sessions were performed, initially 2–3 ECT treatments per week. Subsequently, ECTs were performed less frequently (first once per week; then once a fortnight) in order to train motor skills and maintain mobility. Until the ninth ECT the progress in muscular activity allowed the patient to perform movements with increasing complexity. Progress continued with the patient gaining progressively more function, being able to eat without help, and managing all activities of daily living in the perimeter of his room with little help. Until the 25th ECT he was able to walk without help. Relapse occurred after a while, with great symptom fluctuation, dependent on circumstances, but muscular activity remained better than at admission. ECT was performed with increasing intensity until a maximum of 1152 mC in order to reach a seizure of at least 30–40 s. CGI-I after ECT was rated: 2 (much improved). The long term nominal CGI-I was rated: 3 (minimally improved).

### Repetitive transcranial magnetic stimulation (all variants)

We identified five articles in that TMS/rTMS was performed in FW affecting limb(s) (Table 2 for characteristics) No articles were identified reporting theta-burst TMS for the treatment of FW. Four articles were published in peer-review journals (Schönfeldt-Lecuona et al., 2006; Chastan and Parain, 2010; Gaillard et al., 2012; Broersma et al., 2015), and a fifth article was retrieved from a conference abstract (Kresojevic et al., 2010). Three articles reported single patients (case reports or case series); only two articles included a larger sample [*n* = 70 in Chastan et al. (Chastan and Parain, 2010) and *n* = 11 in Broersma et al. (2015)].

#### Case description

The study by Schönfeldt-Lecuona et al. (2006) had a prospective design and a clearly defined stimulation protocol based on a biological and functional-anatomical etiological hypothesis (see Discussion). This open-label, non-placebo controlled trial reported four patients (3 FW, 1 malingerer). Patients received 2 weeks (5-sessions/week) of rTMS in supra-threshold intensity [110% resting motor threshold (MT)] over the contra-lateral motor cortex to the paralyzed limb with a focal figure-8 coil and 4000 stimuli per day. Thereafter, once the patient started to independently perform own movements of the fingers, sub-threshold rTMS (90% MT) was continued for 4–12 weeks with otherwise the same parameters depending on clinical needs. In all three FW patients rTMS caused a marked amelioration of symptoms over time that was sustained at 1-year follow-up. The estimated nominal group-CGI-I after rTMS was rated (for the 3 FW patients): 2 (much improved). The long term CGI-I was sustained after 1 year at the same level (Table 2 for case related retrospective calculated CGI-I score).

Gaillard et al. (2012) described the case of a 33-year old man who had developed a quadriplegia (and anasthesia, December 2004) 6 months before admission. rTMS was performed at 1 Hz frequency (intensity and coil type not stated) targeting the motor cortex, right and left, over the arm-hand area, and right and left over the legs' cortical motor area (1000 pulses over each region) with a total of 4000 pulses per day. Initially, treatment was applied five times a week for 8 weeks, and thereafter twice in a week. The authors reported marked, progressive symptom amelioration, so that he was able to walk again. A further deterioration led to a new rTMS treatment leading again to symptom amelioration (not otherwise specified), but the patient was then mobile only with a wheelchair. A third deterioration led to phlebitis, pulmonary embolism and pressure soars, and ECT was performed (the ECT performed in this case was illustrated above). CGI-I after the first rTMS series was rated: 2 (much improved). The long term CGI-I was rated: 4 (unchanged in relation to rTMS beginning).

Kresojevic et al. (2010) presented two cases treated with rTMS. One of them (24-year old man) was suffering from a FW (hemiparesis). This patient was treated in a single session with 12 single pulses using a round coil (at initially 30% maximal stimulator output intensity and increasing intensity in 10% steps up to a maximum of 80% stimulator output) over the vertex. The response to rTMS was stated as immediate (“the patient was able to walk again independently”). At 6-months follow-up, a partial deterioration occurred, but he was still able to walk and minor walking difficulties did not influence his daily activities. The patients' CGI-I after TMS was rated: 2 (much improved). The long term CGI-I after 6 months follow-up was rated: 3 (minimally improved).

Chastan et al. (Chastan and Parain, 2010) presented a retrospective analysis of medical records of 70 FW patients (26 male), who had received TMS. Fifty-seven percent of the patients had paraplegia, 37% had a monoplegia, 3% had a tetraplegia, and 3% a hemiplegia. The stimulation protocol was variable. The TMS was principally used for routine diagnostic purposes in each patient. An average of 30 pulses were delivered at about 0.2–0.25 Hz with a circular coil and an intensity of 100% of maximum stimulator output over the motor cortex (“opposite the correspondence paralysis or on both sides for bilateral paralysis,” not otherwise specified). Another session of 30 pulses was sometimes added a few minutes later in case of incomplete improvement. TMS was very effective in 62 patients, with a dramatic improvement in nine, a total recovery in 53 (immediately in 43 patients, within minutes or hours in eight patients, within days in two patients), mild improvement in five, and no effect in three patients. Acute onset of FW was associated with a better outcome (but not age, gender or co-morbid psychiatric disorder). CGI-I after TMS was rated for each reported patient group: 1 (very much improved) for the majority of the FW patients (*n* = 53; 76%); 2 (much improved) for nine patients (13%); 3 (minimally improved) for five patients (7%); and 4 (unchanged) for three patients (4%). Five to six months after TMS, recurrence of FW occurred in eight patients, six of whom were re-stimulated and responded to TMS. There was not sufficient information to calculate long-term CGI-I.

Broersma et al. (2015) presented the first study using a placebo-controlled cross-over design and reported 11 patients with FW with at least a flaccid hand paralysis treated with rTMS. Based on the stimulation parameters proposed by Schönfeldt-Lecuona et al. (2006), active rTMS was delivered with a figure-of-8 coil at 15 Hz over the motor cortex contralateral to hand paralysis (targeting being guided under neuro-navigation in seven patients) during 30 min once a day, for a total of 10 working days within 2 weeks (Table 2 for detailed parameters). The placebo condition consisted of small electrical currents applied with a real electromagnetic device (REMP) placed in front of the magnetic coil at otherwise identical parameters. In the study design, the authors attempted to exclude any other additional therapeutic influences that could result from suggestion or afferent feedback due to rTMS-related supra-threshold muscle contraction. To achieve this goal the authors performed the active condition at an intensity of 80% of MT, and the communication with the patients was limited as much as possible. The stimulation condition switched between active and sham after the first 2 weeks of stimulation with a wash-out phase of at least 2 months between both conditions. Because of dropouts, 11 patients received active rTMS and only eight patients received sham rTMS. The primary outcome measure was an objective change in muscle strength as measured by dynamometry after treatment. The secondary outcome measure was the subjective change in muscle strength after treatment. In patients who received both treatments, active rTMS induced a significantly larger median increase in objectively measured muscle strength (24%) compared to sham rTMS (6%). Eight out of 11 patients receiving active rTMS showed an improvement of at least 20% of muscle strength. However, subjective ratings showed no statistical difference between treatments, i.e., patients did not really perceive these objectively measured motor improvements. As the patients' muscle strength improved, the authors suggested that rTMS alone could potentially improve muscle weakness in FW. However, patients did not report subjective improved functioning of the affected hand, which Broersma et al. interpreted as an indication that decreased muscle strength is not the core symptom in FW. They thus propose that rTMS should be applied as add-on therapy to behavioral approaches in FW. There was not sufficient information to calculate nominal CGI-I scores.

### Nominal CGI-I score reconstruction

For patients treated with rTMS we retrieved sufficient information from the physician-estimated functional changes reported in the manuscripts by Schönfeldt-Lecuona et al. (2006) (*n* = 3), Chastan et al. (*n* = 70) (Chastan and Parain, 2010), Kresojevic et al. (2010) (*n* = 1), and Gaillard et al. (2012) (*n* = 1) that allowed the assessment of 75 FW patients. In the study by Broersma et al. (2015) the main outcome parameter was muscular strength changes assessed by dynamometer. The authors reported that patients were assessed neurologically for sensory deficits, coordination, reflexes and muscle strength at the beginning and end of rTMS treatment. However, the narratives provided in the paper did not allow an estimation of nominal CGI-I scores. Therefore, these patients (*n* = 11) were excluded from the analysis. For patients treated with ECT we retrieved information from the narratives in the articles by Giovanoli (1988) and Gaillard et al. (2012) that allowed us to assess the two patients reported.

For the patients treated with rTMS the estimated scores showed a satisfactory improvement at the short-term: nominal CGI-I scores were 1 (very much improvement) in 54 of 75 patients (72%) and 2 (much improvement) in 13 patients (17%). Only five of the treated patients (7%) improved minimally (CGI-I = 3), and 3 (5%) remained unchanged (CGI-I = 4). Overall, about 88% of these patients improved markedly (very much or much improvement) after stimulations. In no case did patients worsen in relation to rTMS treatment, and no serious adverse event was reported. A long-term CGI-I could not be estimated for the largest study by Chastan et al. (*n* = 70) (Chastan and Parain, 2010). FW symptoms recurred in eight patients 5–6 month after rTMS. In 62 patients treatment seem to have caused some amelioration compared to baseline (not stated). In *n* = 3 cases by Schönfeldt-Lecuona et al. follow-up CGI-I showed a sustained amelioration (Schönfeldt-Lecuona et al., 2006), while in the case by Gaillard the estimated long-term CGI-I was rated 4 (Gaillard et al., 2012). Patients treated with ECT showed a satisfactory response at short-term follow-up as well (ranging CGI-I = 2), but a deterioration of FW symptoms at long-term follow-up (ranging CGI-I = 3 in both cases; Table 2).

## Discussion

### Discussion of literature search results

We concentrated our search exclusively on limb weakness and limb paralysis, since other forms of FNS (such as impaired coordination or balance, dystonia, tremor, myoclonus, fainting, tics, hemiballismus, chorea, parkinsonism, bizarre gait, astasia, abasia, aphonia, swallowing difficulty, urinary retention, loss of touch sensation, double vision, blindness, and deafness) might have a different neurobiological etiology and probably other functional-anatomical correlates (Ejareh Dar and Kanaan, 2016). For this reason, we speculate that differential effects of NIBS methods might come into play when treating different forms of FNS.

The literature search identified two case reports with ECT as treatment for FW (Giovanoli, 1988; Gaillard et al., 2012), five articles with TMS (Schönfeldt-Lecuona et al., 2006; Chastan and Parain, 2010; Kresojevic et al., 2010; Gaillard et al., 2012; Broersma et al., 2015), and none for tDCS, with a total of 86 patients. All identified cases and studies reported a short-term symptom improvement. However, any assumption of a beneficial effect of NIBS in FW has to be seen with caution, as the supporting literature is very sparse and the quality of the small number of identified articles was poor. Major concerns when examining the efficacy of NIBS in FW include the heterogeneity of studies with regard to design and stimulation parameters (paragraph below for more information), the absence of randomized controlled conditions in all but one trial, and the fact that the current literature does not allow a meta-analysis of outcome data. Most of the included studies were case reports or case series (5 out of 7).

### Study designs, parameters, and outcomes

For TMS, only one study by Broersma et al. used a prospective, placebo-controlled, cross-over design with an objectively measured outcome using a dynamometer (Broersma et al., 2015). All other identified trials had no sham condition and only used an unstructured physician oriented clinical impression as outcome measure. The study with the hitherto largest sample of 70 patients by Chastan et al. (Chastan and Parain, 2010) was based on a retrospective sample analysis. Moreover, patients were mostly stimulated for diagnostic purposes and about 60% of the patients were children or adolescents. In all studies, FW symptoms and illness duration of the reported patients were heterogeneous and data were insufficient for a retrospective re-analysis, which would have allowed symptom clustering and meta-analysis. In the five identified articles (Schönfeldt-Lecuona et al., 2006; Chastan and Parain, 2010; Kresojevic et al., 2010; Gaillard et al., 2012; Broersma et al., 2015) no detailed information was presented regarding the way patients were informed and the treatments explained. The magnitude of the effect of the explanatory model could therefore not be estimated. The therapeutic effect of the active rTMS in Broersma et al. (2015) was smaller than the one reported by others; the mean increase of muscular strength was only about 20% (dynamometer), but there was no subjective amelioration of symptoms. The stimulation intensity in that study was deliberately kept at 80% MT, and therefore did not trigger any muscle contractions. This may indicate the importance of the patient becoming aware of movement and intact motor pathways as part of subjective symptom improvement. Placebo effects are likely to be involved in the mechanism of action, since in the study of Broersma, six out of nine patients showed a slight improvement after sham rTMS.

The stimulation protocol and parameters used differed considerably between studies. While most studies used low-frequency stimulation (1 Hz or less), Schönfeldt-Lecuona et al. (2006) and Broersma et al. (2015) delivered rTMS with 15 Hz, considered for rTMS to be high-frequency. Most therapeutic rTMS were performed in a single session, but the studies by Schönfeldt-Lecuona et al. and Broersma et al. applied a longer protocol (over weeks).

### Estimated functional improvement

Despite some limitations, we retrospectively managed to judge the efficacy of the investigated stimulation methods (rTMS and ECT) for treating FW patients using the principles of the CGI-I scale. In total, we identified 88 patients with FW affecting limbs that received either rTMS or ECT). For the cases in which a nominal CGI-I could be retrospectively estimated (*n* = 77) about 90% of them improved markedly (very much or much improvement) after stimulations. Only a minority of the treated patients improved minimally or remained unchanged. In no case did patients worsen significantly after treatment and no serious adverse events were reported. At follow-up, symptom improvement was not quantifiable in terms of CGI-I for the majority of the cases (Table 2 for detailed information).

### ECT vs. rTMS

To our knowledge only two cases of ECT treatment in FW of limbs have been published (Giovanoli, 1988; Gaillard et al., 2012) since this technique was established in psychiatry many decades ago. Both published cases reported dramatic improvements of limb movement related to the ECT, thus causing a great improvement of activity of daily living. Besides the known favorable effects on brain function in major depression, no specific mechanism of action has been elucidated for ECT in relation to FW symptoms. One may speculate that the possible mechanism for short term gains is the reduction of stress due to the amelioration of psychological precipitating factors and an improvement in mood after ECT. A major role of a placebo effect in both described cases accounting for the symptom improvement cannot be ruled out. On the other hand, in both cases improvement was not sustained over time, and both patients had a partial relapse after a while. None of the cases postulated or tried a continuation or maintenance ECT, which is recommended for the treatment of major depression when acute ECT effects do not persist (Petrides et al., 2011). A disadvantage of the ECT might be the economical aspect compared with other NIBS methods; the costs of the general anesthesia and the required specialized personal are included. Given the risks of the general anesthesia and (reversible) post-treatment cognitive disturbances, restriction of ECT to the severest and treatment-resistant FW cases should be considered.

Most of the included articles were related to *TMS/rTMS* (*n* = 5). rTMS may be the NIBS method that is most appropriate for the use in limb FW for different reasons: (1) rTMS can acutely provoke a muscular contraction or transient movements without needing patients cooperation (or intention) to move. (2) rTMS is relatively easy to apply in FW. This is in contrast to stimulations outside the motor cortex for other indications, in which localization strategies for coil positioning are needed (Schönfeldt-Lecuona et al., 2005). The magnetic pulses applied using intensities above MT (supra-threshold stimulation) will trigger a visually noticeable muscle contraction. Because motor pathways are intact in FW, this technique allows targeting the desired motor area with sufficient precision (Herwig et al., 2001, 2003). (3) Longer lasting rTMS causes plasticity changes in brain areas directly under the magnetic coil (Karabanov et al., 2015), but also trans-synaptic changes in areas far from the stimulation site (Strafella et al., 2003, 2004; Pogarell et al., 2007). (4) rTMS is mostly well tolerated, and has no adverse effects if performed within safety limits (Rossi et al., 2009). rTMS is considered not to be painful (depending on the intensity, frequency and train length of trains applied). (5) rTMS treatment is currently performed by physicians, but can also be performed by trained allied medical professionals (nurses, technicians, psychologists). It does not require any anesthetic, and can be performed in an outpatient setting. (6) The costs per session are lower than ECT, and rTMS devises are common nowadays in neurology and psychiatry departments, and in rehabilitation clinics in many high-income countries.

### Why might rTMS work?

#### Psychological aspects

A crucial effect of supra-threshold TMS/rTMS in contrast to other NIBS methods is the patient's conscious perception of the externally triggered movements of their paralyzed limb. This phenomenon is experienced by all patients treated with rTMS, since prior to every therapeutic trial, single-pulse TMS with different intensities will be applied over the motor cortex in order to establish individual MT. In contrast, tDCS can modulate the excitability of cortical networks but does not directly produce action potentials on stimulated networks, and is therefore unable to trigger muscle contractions. Using ECT in FW, limb movements are actually provoked through the induced seizures, but the patient is not capable of noticing them because ECT is performed under general anesthesia. The patient's awareness of the muscle contractions due to TMS may help through a psychological mechanism: depending on the information received, patients become aware of normal function of neuromuscular structures. In addition, TMS triggering of muscle contraction might make patients aware of the possibility of regaining function. All identified studies showed an excellent response to TMS, except the one by Broersma et al. It was in that study that sub-threshold intensity rTMS was used, which does no provoke a muscular contraction. In addition, rTMS bears a high technical and methodological complexity in terms of technical approaches and calibrating steps that have to be performed prior to the therapeutic application, especially when MRI-guided localization techniques for coil positioning are used. Thus, TMS may generate a placebo effect, which in turn helps the patient to recover function immediately after stimulation. The response to rTMS may also be influenced by the information received and by the style which was used to inform the patients about the treatment strategy and purpose.

#### Neurophysiological aspects

An increasing body of literature data suggest that focal functional abnormalities in central networks that control motor cortex activity may play a role in the etiology of FW (Geraldes et al., 2008; Liepert et al., 2008, 2009, 2011). The most convincing hypotheses to explain FW affecting limbs include (i) deficient processing of either motor intention or disruption between motor intention and motor execution or (ii) an overactive self-monitoring with enhanced limbic neural activity, which interferes with movement planning, and initiation within frontal regions and thereby disrupting motor execution (Voon et al., 2011). Studies using functional-imaging methods in patients with FW demonstrated enhanced neural activity within the anterior cingulate area or orbito-frontal cortex and reduced neural activity within prefrontal motor areas during movement execution of the paralyzed limb (Marshall et al., 1997; Spence et al., 2000; Stone et al., 2007). These abnormal activation patterns have been interpreted to reflect an active, but unconscious inhibition of movement planning and execution. Focused rTMS protocols with appropriate stimulation parameters might be able to reverse cortical dysfunction and restore activity in suppressed cortical motor areas (Schönfeldt-Lecuona et al., 2003, 2006; Chastan and Parain, 2010; Nielsen et al., 2013). The stimulation site for rTMS in FW is usually obvious with the primary motor cortex being the most plausible candidate region. However, the most challenging issue is still the choice of stimulation protocol (frequency, intensity, inter-train intervals, duration, and number of daily sessions) that can provoke a lasting positive change in cortical network activity. With regard to the question whether complementary stimulations in other cortical regions than the primary motor cortex could enhance therapeutic efficacy of rTMS in FW, no studies could be found.

#### Neuromodulatory aspects

Single-pulse TMS with short protocols (e.g., performed in only one session for measuring MEPs for diagnostic purposes) might not be causing a durable change in cortical activity. Long-lasting changes and thus changes in cortical neuro-plasticity might only be induced performing longer protocols (e.g., for one or more weeks) using high-frequency (>1 Hz) or low-frequency (≤1 Hz), thus leading to long-term potentiation- or long-term depression-like changes respectively (Pascual-Leone et al., 1994; Chen et al., 1997; Fitzgerald et al., 2006). In most identified trials using TMS in FW, cortical stimulations were performed using repetitive spTMS with frequencies ≤1 Hz for a very short time (<2 or 3 min; Chastan and Parain, 2010; Kresojevic et al., 2010; Gaillard et al., 2012). Therefore, no long-lasting cortical effects could be expected, nor any stable changes in motor function due to the used protocols. The immediate and mostly sustained positive responses to the stimulations must therefore have other reasons. Only few studies used rTMS protocols that might potentially cause plasticity changes. We need to stress that not all of these hypotheses of the mechanisms of action of rTMS have been translated into proven clinically relevant changes, and more research is needed to be sure if they have any clinically meaningful effect (McWhirter et al., 2015). Furthermore, given that rTMS has existed as a technique since the 1990s, the number of trials published in this field is amazingly low. Publication bias could be a partial explanation for this, as may be the paucity of clinicians considering rTMS in rehabilitation neurology. However, first rTMS therapeutic trials have been performed to relieve other forms of FNS such as dystonia, myoclonus, tremor, parkinsonism, stereotypies, non-epileptic seizures, functional aphonia, or sensory or visual loss (Chastan et al., 2009, 2011; Dafotakis et al., 2011; Saha et al., 2011; Garcin et al., 2013; Parain and Chastan, 2014; Shah et al., 2015), mostly yielding to symptom amelioration.

## Recommendation in evidence-based guidelines and future study designs

In 2014 evidence-based guidelines on rTMS were published and included recommendations for “Motor Conversion Disorders” in general (Lefaucheur et al., 2014). The degree of recommendation on the efficacy of rTMS for motor conversion disorders was: “No recommendation for low or high frequency rTMS of M1 or delivered at the vertex, using a focal or a non-focal coil” (Lefaucheur et al., 2014). The Cochrane library has published recommendations for TMS in the treatment of schizophrenia, depression, obsessive-compulsive disorder, amyotrophic lateral sclerosis, epilepsy and post-traumatic stress disorder (http://onlinelibrary.wiley.com/cochranelibrary/), but did not address the topic of FW. Regarding ECT or tDCS, no sources were identified that reported evidence-based recommendations for its use in FW or more generally in FNS. Consistent with previous recommendations for the publication of case reports or case series (Lepping et al., 2007), and to allow clinically meaningful analyses from case series, we recommend that the following information should be included in any publication:

1. Regarding devices and targeting procedure: the type of coil, the type of stimulator, the type of pulse waveform, the definition of the target and of its localization method, including the type of navigation system (if used), and the orientation and angle of the coil. Sham rTMS should be performed using original sham coils. Other alternatives should be accurately described and the rationale for the chosen technique should be highlighted.
2. Regarding stimulation parameters: the intensity of the stimulation according to MT (resting/active MT) or maximal stimulator output, the frequency and duration of rTMS trains, the duration of the inter-train interval, the number of trains applied, the total number of rTMS pulses per session, the duration of each session, the number of sessions, the duration of the interval between the sessions, and the total duration of the treatment. The rationale for the chosen treatment protocol should be stated.
3. Regarding ratings for motor symptoms and quality of life: the outcome assessment should be at least performed using CGI-I scores. Limb muscular strength should be assessed for each muscular group (force rated from 0 = complete paralysis to 5 = normal strength) at the beginning of treatment and at follow-up (if possible 6 and 12 months after cessation of treatment). Objective assessment of muscle force using dynamometers and of quality of life, using validated questionnaires are desirable. Raters should be blinded to the stimulation condition. In addition to objective ratings, the assessments of the treatment efficacy should include subjective ratings of symptom severity, as there may be a disparity between the patient's and the doctor's rating.
4. Regarding the explanatory information for patients, the information received by the patient about the rTMS procedure and its expected positive or adverse effects should be outlined. The given information should be objective. Explanatory information for patients are not yet standardized and from a therapeutic perspective, its effect magnitude on clinical symptoms is unknown.
5. Regarding a control condition, placebo-controlled study designs would be highly desirable. However, investigators should be aware that patients who are not treatment-naïve would easily detect the difference between the two conditions (particularly due to the perceptible scalp sensations by active stimulation). Therefore, except when using special placebo coils that provoke scalp effects similar to an active stimulation [as in Broersma et al. (2015) and Rossi et al. (2007)], we suggest that future studies should either be designed as parallel-arm studies (avoiding sham detection in a cross-over design) or as head-to-head studies, comparing active rTMS with usual therapeutic management of FW.

## Conclusion

The results of our systematic review provide preliminary evidence that NIBS methods, especially motor cortex rTMS, may be beneficial in the treatment of conversion weakness and paralysis. Most included rTMS studies reported acute beneficial effects on limb function despite heterogeneous protocols. In particular, the crucial influence of an externally triggered muscular contraction should be emphasized. Further rTMS trials should include a control condition, a greater number of sessions, and longer stimulation protocols with proven lasting effects on cortical excitability. However, although advances have been made in the last few years both in diagnostic methods and in the groundwork for a neurobiological model of FW, no definitive rationale for stimulation parameters and for the optimal setting is available. Therefore, further basic research in this area is needed (Aybek et al., 2008). Probably due to practical aspects the future of ECT in this area is expected to be less promising than rTMS. Despite this practical advantage, it remains to be demonstrated that rTMS can have a real therapeutic benefit in the long term, and any impact on the neural mechanisms of FW beyond merely inducing psychological or non-specific placebo effects. Our systematic review contributes to the current knowledge of rTMS application in the treatment of FW, updating the reviews previously published by Pollak et al. (2014) and Parain and Chastan (2014). In summary, the available evidence to date suggests that the application of NIBS in FW is feasible and beneficial. However, due to the small number of published cases in open-label studies, this conclusion should be considered with caution.

## Author contributions

CS, MG, PL made substantial contributions to conception and design of the review, CS, J-PL, MG performed the literature-search, analyzed the data, and wrote the manuscript. DN, AS, JL participated in drafting the manuscript, wrote the manuscript, and revisited it critically. BC made substantial contributions to the conception of the review, and revisited the manuscript critically. All authors gave the final approval of the version to be published and agreed to be accountable for all aspects of the work in ensuring that questions related to the accuracy or integrity of any part of the work are appropriately investigated and resolved.

### Conflict of interest statement

The authors declare that the research was conducted in the absence of any commercial or financial relationships that could be construed as a potential conflict of interest.
